# Mendelian Randomization Analyses Reveal the Causal Effect of Cathepsin Z on the Risk of Different Subtypes of Thyroid Cancer

**DOI:** 10.1155/ije/9916769

**Published:** 2025-10-10

**Authors:** Shao-Kun Sun, Jian-Liang Yuan

**Affiliations:** Department of Thyroid Surgery, The First People's Hospital of Kunshan, Suzhou, Jiangsu, China

**Keywords:** cathepsin Z, causal relationship, Mendelian randomization, thyroid cancers

## Abstract

**Background:**

Earlier research studies employing observational methods have suggested a possible relationship between the activity of cathepsin Z and thyroid cancer (TC). However, the causal relationship linking the cathepsin Z to TC has yet to be fully established, especially for different subtypes of TC.

**Methods:**

The study employed accessible genomewide association study (GWAS) datasets to conduct bidirectional Mendelian randomization (MR) analyses. The primary approach for conducting MR analysis was the application of inverse variance weighting (IVW).

**Results:**

The MR analysis indicated that elevated cathepsin Z levels are positively linked to an elevated risk of papillary TC (PTC) development. In contrast, reverse MR indicated that PTC cannot contribute to increasing cathepsin Z levels.

**Conclusion:**

Our MR analysis suggests a causal role of cathepsin Z in the development of PTC, offering valuable insights for future mechanistic studies and potential clinical applications targeting cathepsin-mediated pathways in cancer.

## 1. Introduction

Thyroid cancer (TC) represents the most common malignancy of the endocrine system, and its global incidence continues to rise [1]. While the majority of TC has 5-year survival rates nearing 98.5% [2], the disease still imposes a significant financial burden on patients [3]. Furthermore, early metastasis to central lymph nodes is frequently seen in papillary TC (PTC) and follicular TCs (FTC) [4]. This factor contributes to poor prognosis and remains a leading cause of mortality among TC patients. Consequently, investigating the molecular mechanisms underlying the development of TC is imperative, with the aim of enhancing both the prognosis and the quality of life for patients afflicted with this condition.

Over the past few years, the critical role of cathepsins in the advancement of cancer has been underscored by numerous studies, particularly in tumor invasion and metastasis. Cathepsins participate in several essential physiological processes, including tissue remodeling, proteolysis, and apoptosis [5, 6]. Cathepsin Z, a cysteine carboxypeptidase, is found mainly in immune cells such as macrophages, monocytes, and dendritic cells [7]. Accumulating evidence links elevated cathepsin Z to several cancers, colorectal, gastric and prostate, and suggests it drives tumor progression by disrupting processes such as proliferation and invasion [8, 9]. However, the interplay between cathepsin Z and the development of different subtypes of TC has been explored in little observational investigations or clinical trials. Previous studies have established that heightened levels of cathepsin Z are associated with an increased risk of TC [5]. However, the biological roles attributed to cathepsin Z seem to differ substantially among the diverse TC subtypes.

Mendelian randomization (MR) utilizes genetic variance as the instrumental variable (IV) to investigate causal relationships [10]. Two-sample MR studies, in particular, minimizes confounding by using genetic variants such as single nucleotide polymorphisms (SNPs), which are largely unaffected by environmental influences. This methodology confers a significant advantage over conventional observational research studies by reducing the risk of confounding bias. In the field of oncology, MR analysis sheds light on the intricate relationships between risk factors and oncogenesis, offering valuable insights for the establishment of strategies for prevention and treatment within the clinical domain [11].

In the present investigation, comprehensive data encompassing cathepsin Z and TC were sourced from an extensive genomewide association study (GWAS). Subsequently, a two-sample MR analysis was executed, complemented by a reverse MR approach to adjust for pleiotropic influences of genetic variants and to mitigate the impact of potential confounding factors. This research aims to elucidate the causal relationship between cathepsin Z and the etiology of TC and further investigates its relationship with specific histological subtypes.

## 2. Materials and Methods

### 2.1. Study Design

Using a two-sample MR analysis, we examined the causal relationship between cathepsin Z and different subtypes of TC. MR relies on genetic variation as an IV to estimate the causal link between a specific exposure and the outcome under investigation. For IVs to be valid in an MR analysis, they must meet three essential assumptions: (1) IVs must exhibit a robust correlation with the exposure of interest; (2) IVs must be independent of any confounding factors; and (3) IVs must exert their influence on the outcome exclusively through the exposure, ruling out any alternative pathways.

### 2.2. Data Source

Genetic instruments for measuring the concentrations of cathepsin Z were derived from the INTERVAL study, encompassing a cohort of 3301 individuals of European ancestry [12]. Every patient offered the informed consent, and the study's ethical clearance was granted by the National Research Ethics Service (11/EE/0538). Summary-level data are available at https://gwas.mrcieu.ac.uk. Aggregate statistical data for TC, comprising log odds ratio (OR) estimates and standard errors for instrumental SNPs, were retrieved from the FinnGen database (https://r5.finngen.fi/). The dataset comprised 1783 TC cases and 287,137 controls. In addition, the database furnished correlations between instrumental SNPs and PTC and FTC, encompassing 1386 cases of PTC and 149 cases of FTC.

### 2.3. Selection of Instrumental Variables

The identification of cathepsin-related IVs for MR analyses adhered to the following criteria: (a) an r2 measure of linkage disequilibrium < 0.001 within a 10,000 kb window, (b) *p* values below the genomewide significance threshold (5 × 10^−6^), (c) minor allele frequency (MAF) > 0.01, and (d) an F-statistic > 10 was used to indicate sufficient instrument strength.

### 2.4. Statistical Analysis

The common two-sample MR methods involve the random effects inverse variance weighted (IVW) method, MR–Egger, and the weighted median estimator.

The IVW approach serves as the principal method for evaluating the relationship of causality between exposure and outcome between exposure and outcome [13]. At the same time, MR–Egger and the weighted median estimator are utilized as validation methods. OR and 95% confidence intervals (CIs) were employed to assess whether a causal link exists between cathepsins and cancer risk. MR–Egger works by performing regression analysis between the effect estimates of IVs and their associations. In this way, whether IVs are related to confounding factors can be determined [14]. The weighted median involves estimating the effect of each IV and weighting them based on their precision, typically using the inverse of their variance. The weighted median of these effects is then calculated to produce an overall effect estimate [15]. Cochran's *Q* test was employed as a sensitivity analysis to check for heterogeneity, with *p* value > 0.05 suggesting no significant heterogeneity [16]. MR–Egger incorporates the intercept to evaluate pleiotropy, where an intercept *p* value below 0.05 signifies the occurrence of horizontal pleiotropy [11]. MR-PRESSO can detect horizontal pleiotropy among all IVs and identify and remove outlier SNPs. It reduces the impact of horizontal pleiotropy and enhances the accuracy of causal inference [17].

A reverse MR analysis was conducted, treating TC as exposures and cathepsins as outcomes, to investigate whether cancer exerts a causal influence on cathepsins, as suggested by the forward MR analysis.

MR analyses were conducted using the “TwoSampleMR” package in R (Version 4.4.1). A *p* value < 0.05 was considered statistically significant.

## 3. Results

### 3.1. MR Main Analysis Results

In order to examine how cathepsin Z influences the likelihood of developing TC, PTC, and FTC, two-sample MR analyses were conducted for cathepsin Z, assessing both the influence on TC and discerning the influence on PTC and FTC. Results from the MR analysis showed that increased concentrations of cathepsin Z correlated positively with an increased susceptibility to PTC (*p*=0.011, OR = 1.170, 95% CI = 1.036–1.322) (Table 1).

Both MR–Egger intercept and MR-PRESSO global tests indicated no significant signs of directional pleiotropy concerning these causal relationships, as shown in Table 2.

### 3.2. Reverse MR Analysis Results

In these reverse MR analyses, cancer was classified as the independent variable, with cathepsin Z serving as the dependent variable, and cancer-associated SNPs (*p* < 5 × 10^−5^) were used as IVs to examine whether the significant findings from the forward analysis could be explained by reverse causality.

The results shown in Table 3 provided no evidence of reverse causality between cathepsin Z concentrations and the likelihood of developing PTC. In addition, the *p* values for both MR–Egger intercept (0.334) and the MR-PRESSO global test (0.804) indicated no evidence of directional pleiotropy (Table 4).

## 4. Discussion

In the present investigation, a systematic analysis was conducted to explore the potential causal links between cathepsin Z enzymes and the risk associated with TC, PTC and FTC, utilizing genetic IVs. This is the MR analysis with a large sample size based on genetic consortia to explore the causal role of cathepsin Z in different subtypes of TC. From the findings of MR analyses, we recognized cathepsin Z as a notable risk factor for PTC, lacking any indication of a reverse causal relationship involving cathepsin Z.

During the last several decades, we have witnessed a marked increase in the prevalence of TC, resulting in its recognition as a significant global health challenge. In 2022, the number of new TC cases globally is estimated to be more than 820,000, ranking 7th out of 36 cancers, with 206,487 cases in males and 614,686 cases in females [1]. The prevalence of TC within China's population is among the top 10 for both men and women; the incidence rate for women is 49.4 per 100,000 people. In other words, for every 100,000 women, there are close to 50 new cases of TC each year, which is the third most prevalent malignancy in the female population. The incidence rate among men is 17.32 per 100,000, which is the seventh most common cancer among men [18].

Previous studies have highlighted the association between cathepsins and TC. Cathepsin S has been suggested as a potential predictive marker for both the progression and prognosis of PTC [6]. In addition, cystatin E has been documented to exhibit tumor-suppressive properties in a spectrum of malignancies such as cervical, gastric, and renal cancers. However, a study showed that it functions as a promoter of tumorigenesis in TC [19]. In addition, prior research has demonstrated that elevated cathepsin Z levels are linked to a higher TC risk [5].

Cathepsin Z is a lysosomal cysteine protease, which belongs to the C1 family of proteases. Cathepsin Z functions as a cysteine carboxypeptidase and is primarily found in immune cells, including macrophages, monocytes, and dendritic cells, where it serves as a pivotal factor in the modulation of cellular proliferation, maturation, migration, and adhesion [9]. A previous study found that cathepsin Z was linked to survival outcomes in patients with colorectal cancer (Stages I-III). Further analysis revealed that higher levels of cathepsin Z were associated with reduced overall survival in patients who did not receive chemotherapy [8]. Furthermore, kidney tumor cells produce, on average, 5.5 times more cathepsin Z than embryonic kidney cells [20]. Not only that, but the MR study conducted by Peng et al. showed that cathepsin Z increased the overall risk of renal cancer, and cathepsin F was observed to increase the risk of clear cell renal cell carcinoma [21]. In addition, earlier research has shown that cathepsin Z is involved not only in the chronic inflammation of the gastric mucosa but also in the oncogenic processes leading to gastric malignancy. Cathepsin Z levels were observed to be elevated in gastric cancer tissues as opposed to adjacent non-neoplastic mucosa [22]. Deng et al. conducted a study demonstrating that higher concentrations of cathepsin Z corresponded to an elevated risk of developing TC. However, reverse MR analysis did not reveal any significant correlation between TC and cathepsin Z levels [5]. However, in breast cancer progression, the role of cathepsin Z is inconsistent with the above in this paper. Zhou et al. found that cathepsin Z has a protective effect against in situ breast cancer [23]. Li et al. also analyzed the relationship between histones and breast cancer and concluded that altered cathepsin Z methylation levels in peripheral blood may be associated with breast cancer, particularly in young women [24].

Recent evidence has identified cathepsin Z as an important driver of malignant progression. In cancer, it facilitates tumor and endothelial cell adhesion, as well as their migration and invasion through the extracellular matrix. Furthermore, cathepsin Z promotes tumor progression by evading cellular senescence and inducing epithelial–mesenchymal transition [25]. Early research also found that several of the tumor-promoting functions of cathepsin Z were not dependent on its described catalytic activity but instead were mediated via the Arg–Gly–Asp (RGD) motif in the enzyme prodomain, which regulated interactions with integrins and the extracellular matrix [26]. In hepatocellular carcinoma, upregulation of CTSZ was significantly associated with advanced clinical stage, and overexpression of Cathepsin Z contributes to tumor metastasis by inducing epithelial–mesenchymal transition [27].

Previous studies have not thoroughly investigated the potential causality linking cathepsin Z to the development of different subtypes of TC. Two-sample MR analyses were conducted in our research, yielding dependable findings. First, the MR approach is advantageous as it can sidestep potential biases due to reverse causality and confounding factors, while also being more time- and resource-efficient compared to traditional observational studies. Second, reverse MR approaches helped to further reduce reverse causation bias. Third, our study was restricted to individuals of European ancestry to mitigate population stratification bias. Finally, our study included not only TC but also subgroups of it (PTC and FTC), and this gives a fuller picture of the relationship between cathepsins and different subtypes of TC.

While this study possesses notable strengths, it is essential to also consider its limitations. First, this study only assessed the causal effect of cathepsins on cancer risk at the genetic level. Further experimental validation is crucial for confirming causal relationships. We plan to include rigorous experiments in our future research to uncover the biological mechanisms behind the potential causal associations found in this study. Second, even though the study populations were all European, we did not account for within-population structures. This omission might have caused hidden population structures to confound the causal relationship. Third, because the study populations were all European, restricting the study area to Europe may result in potential sample overlap. Fourth, the limited sample size for TC's histological subtypes might result in false-negative errors. Finally, this research population was comprised solely of individuals with European ancestry, which may restrict the applicability of the findings to other ethnic populations. Future MR studies with larger and more diverse sample sizes, as well as randomized controlled trials, are needed to confirm these results.

## 5. Conclusion

In summary, the research, using two-sample and reverse MR methods, suggests a potential causal link between cathepsin Z and PTC. However, caution is critical when interpreting this study's findings, as misinterpretation may lead to erroneous conclusions and impact future research. Further investigations are necessary to confirm these results and consider their suitability for application in clinical research.

## Figures and Tables

**Table 1:** Causal association of cathepsin Z on TC, PTC, and FTC in Mendelian randomization analysis.

Cathepsin Z	SNP	Inverse variance weighted		MR–Egger		Weighted median	
		OR (95% CI)	*p* value	OR (95% CI)	*p* value	OR (95% rCI)	*p* value
TC	13	1.058 (0.946–1.182)	0.323	0.999 (0.836–1.193)	0.988	1.009 (0.880–1.158)	0.895
PTC	13	1.170 (1.036–1.322)	0.011	1.105 (0.912–1.339)	0.328	1.150 (0.989–1.338)	0.070
FTC	13	0.715 (0.495–1.033)	0.074	0.918 (0.517–1.631)	0.776	0.826 (0.521–1.309)	0.416

**Table 2:** Pleiotropy and heterogeneity analyses of two-sample MR between cathepsin Z and different histological types of thyroid cancer.

Exposure	Outcome	MR–Egger intercept		MR-PRESSO global	MR-IVW			MR–Egger		
		Egger intercept	*p*	*p*	*Q*	*Q*_df	*Q*_pval	*Q*	*Q*_df	*Q*_pval
Cathepisn Z	TC	0.018	0.429	0.469	12.804	12	0.383	12.065	11	0.359
	PTC	0.018	0.467	0.671	10.457	12	0.576	9.891	11	0.540
	FTC	−0.080	0.292	0.822	7.559	12	0.819	6.332	11	0.850

**Table 3:** Causal association of TC, PTC, and FTC on cathepsin Z in reverse Mendelian randomization analysis.

	SNP	Inverse variance weighted		MR–Egger		Weighted median	
		OR (95% CI)	*p* value	OR (95% CI)	*p* value	OR (95% CI)	*p* value
*TC*							
Cathepsin Z	100	0.996 (0.972–1.020)	0.733	0.991 (0.955–1.029)	0.648	0.999 (0.953–1.048)	0.968

*PTC*							
Cathepsin Z	111	1.006 (0.982–1.031)	0.612	0.985 (0.936–1.036)	0.551	1.005 (0.968–1.042)	0.809

*FTC*							
Cathepsin Z	21	1.008 (0.982–1.035)	0.559	1.039 (0.977–1.105)	0.238	1.012 (0.976–1.050)	0.515

**Table 4:** Pleiotropy and heterogeneity analyses of two-sample MR between different histological types of thyroid cancer and cathepsin Z.

Exposure	Outcome	MR–Egger intercept		MR-PRESSO global	MR-IVW			MR–Egger		
		Egger intercept	*p*	*p*	*Q*	*Q*_df	*Q*_pval	*Q*	*Q*_df	*Q*_pval
TC	Cathepisn Z	0.002	0.756	0.311	105.572	99	0.307	105.468	98	0.285
PTC	Cathepisn Z	0.007	0.334	0.804	96.887	110	0.810	95.947	109	0.810
FTC	Cathepisn Z	−0.022	0.297	0.695	16.290	20	0.699	15.140	19	0.714

## Data Availability

The datasets generated during and/or analyzed during the current study are available from the corresponding author on reasonable request.
